# Py2T Murine Breast Cancer Cells, a Versatile Model of TGFβ-Induced EMT *In Vitro* and *In Vivo*

**DOI:** 10.1371/journal.pone.0048651

**Published:** 2012-11-07

**Authors:** Lorenz Waldmeier, Nathalie Meyer-Schaller, Maren Diepenbruck, Gerhard Christofori

**Affiliations:** Institute of Biochemistry and Genetics, Department of Biomedicine, University of Basel, Basel, Switzerland; Thomas Jefferson University, United States of America

## Abstract

**Introduction:**

Increasing evidence supports a role of an epithelial to mesenchymal transition (EMT) process in endowing subsets of tumor cells with properties driving malignant tumor progression and resistance to cancer therapy. To advance our understanding of the underlying mechanisms, we sought to generate a transplantable cellular model system that allows defined experimental manipulation and analysis of EMT *in vitro* and at the same time recapitulates oncogenic EMT *in vivo*.

**Methodology/Results:**

We have established a stable murine breast cancer cell line (Py2T) from a breast tumor of an MMTV-PyMT transgenic mouse. Py2T cells display a metastable epithelial phenotype characterized by concomitant expression of luminal and basal cytokeratins and sheet migration. Exposure of Py2T cells to transforming growth factor β (TGFβ) *in vitro* induces reversible EMT accompanied by downregulation of E-cadherin and upregulation of mesenchymal markers, including EMT transcription factors, and a gain in single cell motility and invasiveness. Py2T cells give rise to tumors after orthotopic injection into syngeneic FVB/N mice. Notably, transplantation of epithelial Py2T cells results in the formation of invasive primary tumors with low to absent E-cadherin expression, indicating that the cells undergo EMT-like changes *in vivo*. This process appears to at least in part depend on TGFβ signaling, since tumors formed by Py2T cells expressing a dominant-negative version of TGFβ receptor widely maintain their epithelial differentiation status.

**Conclusions/Significance:**

Together, the data demonstrate that the Py2T cell line represents a versatile model system to study the EMT process *in vitro* and *in vivo*. The observation that Py2T cells give rise to tumors and collectively undergo EMT-like changes *in vivo* highlights the suitability of the Py2T model system as a tool to study tumor-related EMT. In particular, Py2T cells may serve to corroborate recent findings relating EMT to cancer cell stemness, to therapy resistance and to tumor recurrence.

## Introduction

Epithelial to mesenchymal transition (EMT) is an embryonic cellular program during which polarized epithelial cells lose their cell-cell adhesions and convert into a motile mesenchymal cell type [1], [2]. These phenotypic changes can be induced by a plethora of signals, including hypoxia, Wnt signaling, epidermal growth factor (EGF), hepatocyte growth factor (HGF), transforming growth factor β (TGFβ), and many more [3], [4]. Intracellular signaling pathways then integrate these signals to initiate the acquisition of mesenchymal traits via an elaborate network of EMT-related transcription factors [5], culminating in the loss of E-cadherin, a central hallmark of an EMT [6]. In the adult, an analogous program can be reactivated in the setting of solid tumors (termed oncogenic or Type III EMT) [7]. During the last two decades, EMT has been in the focus of many research fields and laboratories [2]. One long-standing interest is based on the concept that EMT of cancer cells facilitates their dissociation from primary tumors and their invasion of surrounding tissue and intravasation, thereby contributing to the initial steps of metastasis [1], [8], [9]. Consistent with the metastatic role of an EMT, recent results have indicated that EMT confers stem cell-like traits to tumor cells [10]–[12]. These results have also provided an attractive explanation for the findings that an oncogenic EMT contributes to resistance against cancer therapy, escape from oncogene addiction and recurrence of tumor growth [13]–[16]. A number of normal and transformed cell lines of murine and human origin have been described and used to study EMT *in vitro*, yet model systems that allow the study of breast cancer EMT both *in vitro* and *in vivo* have remained scarce.

To meet this need, we set out to establish a cellular model of breast cancer EMT that with one cellular system allows the study of epithelial plasticity *in vitro* and of EMT and malignant tumor progression *in vivo*. We here report the establishment of a cell line (Py2T) derived from a primary breast tumor of MMTV-PyMT transgenic mice. Py2T cells undergo EMT *in vitro* upon TGFβ stimulation and, upon orthotopic injection into syngeneic or nude mice, they form primary tumors with an EMT-like phenotype, which is at least in part dependent on the responsiveness of the transplanted tumor cells to TGFβ signaling.

## Results

### Py2T, a Novel Breast Cancer Cell Line Undergoing TGFβ-induced EMT

**Figure 1:**
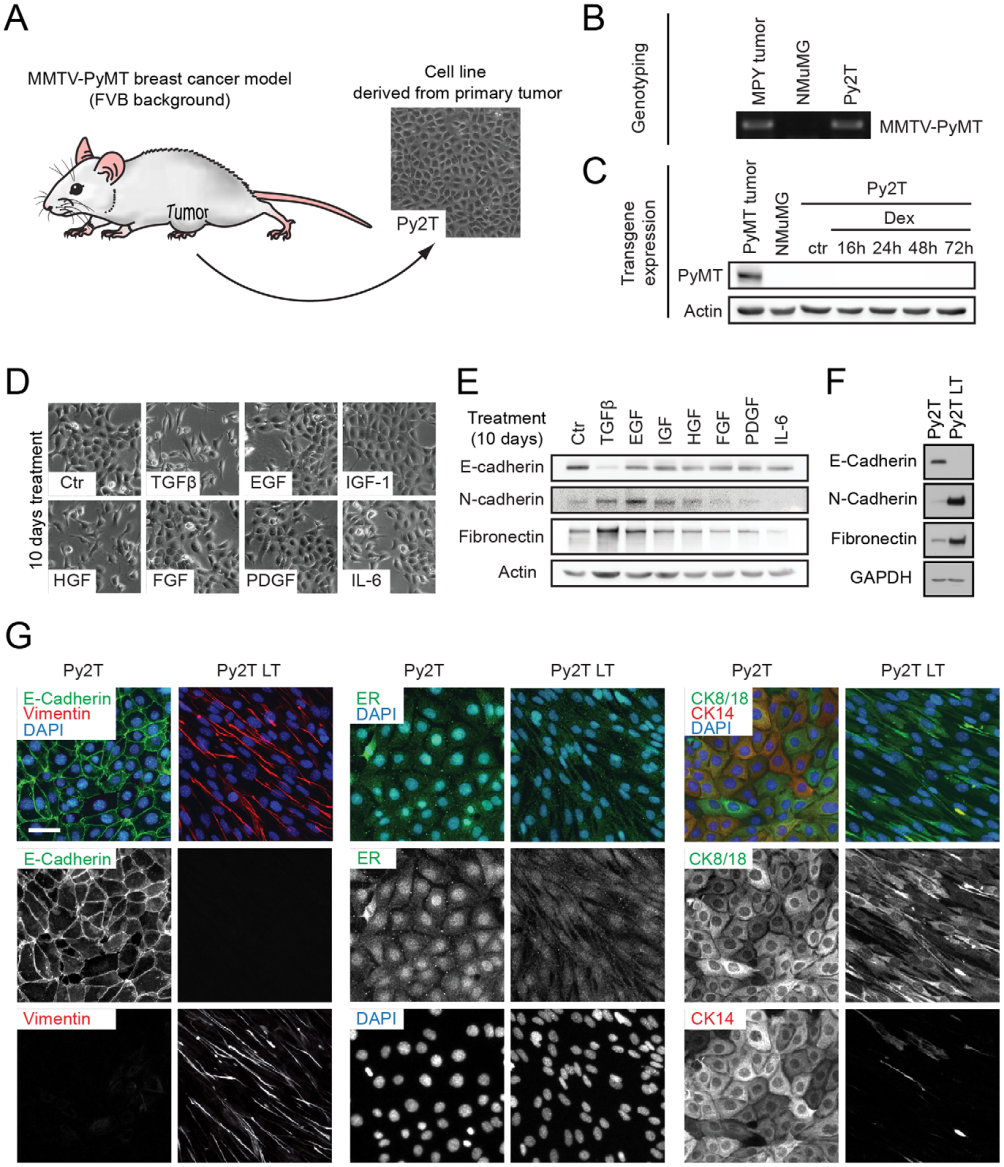
Establishment of a murine breast cancer cell line undergoing TGFβ-induced EMT. (**A**) Primary tumor cells were isolated from an advanced breast tumor of a MMTV-PyMT transgenic female mouse and were cultured for at least 2 months prior to further experimentation, resulting in a novel cell line termed Py2T. (**B**) Py2T cells maintain the MMTV-PyMT transgene. The MMTV-PyMT transgene was detected by PCR and agarose gel electrophoresis. DNA from an MMTV-PyMT tumor and from normal murine mammary gland (NMuMG) cells served as positive and negative controls, respectively. (**C**) Py2T cells lost the expression of the MMTV-PyMT transgene. Immunoblotting for the PyMT protein was performed on lysates of Py2T cells untreated or treated with 0.1 µM Dexamethasone for up to 72 h to induce the MMTV promoter. Lysates of an MMTV-PyMT tumor and NMuMG cells served as positive and negative controls, respectively. (**D**) Treatment of Py2T cells with known EMT inducers. Cells were continuously treated with the indicated growth factors and cytokines for 10 days (2 ng/mL TGFβ1; 50 ng/mL EGF; 10 ng/mL IGF-I; 50 ng/mL HGF; 20 ng/mL FGF-2; 20 ng/mL PDGF-BB; 50 ng/mL IL-6). Potential morphological changes were analyzed by phase-contrast microscopy. (**E**) Expression of epithelial (E-cadherin) and mesenchymal (N-cadherin, fibronectin) markers were analyzed by immunoblotting of the lysates of cells treated in (D). (**F**) Immunoblotting analysis of EMT marker expression in Py2T and Py2T LT cells. The mesenchymal subline Py2T LT (long-term) was generated by TGFβ-treatment of Py2T cells for at least 20 days, and was subsequently maintained in TGFβ containing growth medium. (**G**) Analysis of markers for EMT and breast cell type before and after TGFβ-induced EMT. Immunofluorescence staining was performed with antibodies against E-Cadherin (epithelial marker), vimentin (mesenchymal marker), estrogen receptor alpha (ERα), cytokeratin 8/18 (luminal markers) and cytokeratin 14 (basal marker). Scale bar, 20 µm.

To establish a cellular model system that could be used to study epithelial to mesenchymal transition (EMT) *in vitro* and also *in vivo*, we sought to establish stable cancer cell lines from primary breast tumors. Since EMT is regarded as a prerequisite in the early steps of metastasis, we chose to isolate cells from tumors of the highly metastatic MMTV-PyMT mouse model of breast cancer [18], [19]. After recovery from culture shock and passaging for 2 months, an isolated pool of cells displayed a uniform cobblestone-like morphology typical of differentiated epithelial cells (Figure 1A). We termed this cell line Py2T (Polyoma-middle-T tumor). The presence of the MMTV-PyMT transgene in these cells could be confirmed by genotyping (Figure 1B). Curiously, PyMT transgene expression was not maintained during extended culturing (Figure 1C).

Next, we investigated whether treatment with a selection of known inducers of EMT [3] could induce EMT-like morphological changes in cultured Py2T cells. Both transforming growth factor β (TGFβ) and hepatocyte growth factor/scatter factor (HGF) provoked loss of cell-cell contacts, which was not observed with other treatments, even after prolonged treatment for 10 days (Figure 1D). Interestingly, only TGFβ treatment resulted in a classical “cadherin-switch”, a hallmark of EMT in which expression of the epithelial cell adhesion molecule E-cadherin is lost and expression of mesenchymal N-cadherin is gained [29]. Furthermore, we observed an upregulation of the mesenchymal marker fibronectin only in TGFβ-treated cells and to a lesser extent in EGF-treated cells (Figure 1E). Therefore, among all the factors tested, only TGFβ induced a *bona fide* EMT in Py2T cells.

TGFβ is known to exert cytostatic effects via effector arms downstream of the canonical Smad2/3 pathway in normal cells. However, cancer cells often develop resistance to TGFβ-induced cell cycle arrest [30]. The canonical TGFβ pathway was activated in Py2T cells upon TGFβ treatment, indicated by the nuclear translocation of the Smad2/3 complex and the activation of Smad3 by phosphorylation (Figure S1A). Furthermore, transient transfection of a promoter reporter construct in which firefly luciferase expression was under the control of a Smad-binding element (SBE) revealed a dramatic induction of transcriptional activity upon TGFβ stimulation, while there was no detectable activity in untreated cells (Figure S1B) [23]. Despite an intact canonical pathway, we did not observe any significant increase in cell cycle arrest or apoptosis upon TGFβ treatment of Py2T cells (data not shown).

To establish an experimental system that allowed direct comparison of epithelial versus mesenchymal cells without prior lengthy TGFβ treatment, Py2T cells were treated with TGFβ for 20 days and subsequently maintained as mesenchymal subline (Py2T LT) in growth medium containing TGFβ. Conveniently, Py2T LT cells preserved their mesenchymal phenotype, even when frozen and re-cultured in the presence of TGFβ. As confirmed by immunoblotting analysis, Py2T LT cells displayed a lack of E-cadherin expression, along with high expression of the mesenchymal markers N-cadherin and fibronectin (Figure 1F). Furthermore, immunofluorescence staining against E-cadherin and the mesenchymal marker vimentin were mutually exclusive in Py2T and Py2T LT cells, respectively, further verifying their distinct epithelial and mesenchymal states (Figure 1G *left*).

**Figure 2:**
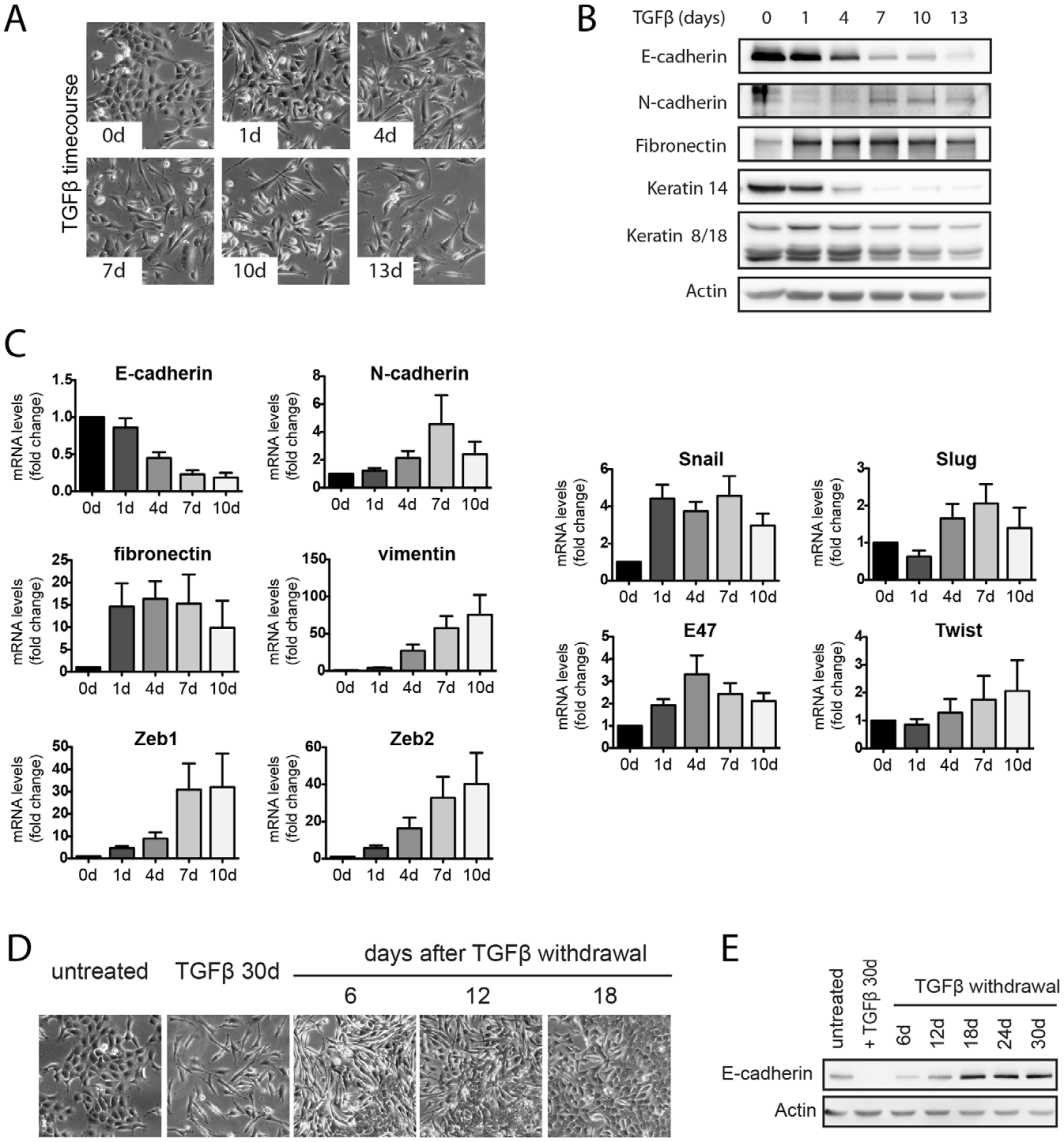
Kinetics and reversibility of TGFβ-induced EMT in Py2T cells. (**A**) Morphological changes of Py2T cells during a time-course of TGFβ-treatment. Cells were cultured in growth medium containing TGFβ (2 ng/ml) and phase-contrast microscopy pictures were taken at the indicated times. (**B**) Immunoblotting analysis of lysates prepared from Py2T cells treated as in (A). The expression of epithelial (E-cadherin), mesenchymal (N-cadherin, fibronectin), luminal (CK8/18) and basal (CK14) markers was analyzed. (**C**) Changes in the expression of EMT markers during TGFβ-induced EMT of Py2T cells. Py2T cells were treated for 10 days with TGFβ as described in (A). RNA was extracted at the indicated time points of TGFβ-treatment and quantitative RT-PCR was performed with primers specific for the EMT markers indicated. Expression levels are shown as mean fold difference of untreated cells (0d) ± S.E.M of 5 independent experiments. (**D–E**) Reversibility of TGFβ-induced EMT. Py2T cells were treated with TGFβ for 30 days to induce EMT and were then further cultured without TGFβ for additional 30 days. Phase-contrast microscopy images were taken at the indicated time points (E). E-cadherin expression levels were analyzed throughout the experiment by immunoblotting (F).

To determine the cell type represented by Py2T cells and to further characterize the effects of TGFβ-induced EMT on cellular identity, we stained for relevant breast cancer and mammary gland cell lineage markers. As the bulk of MMTV-PyMT tumors consist of luminal, estrogen receptor α (ERα)-positive epithelial cells, we expected Py2T cells to display a similar expression pattern. Indeed, we could detect nuclear ERα staining in untreated cells, indicative of luminal differentiation (Figure 1G *middle*). Py2T LT cells however did not stain positive for ERα, consistent with a role of ERα in maintaining an epithelial phenotype and suppressing EMT [31]. To determine whether Py2T cells represent a luminal or a basal mammary gland cell subtype, we stained for luminal cytokeratin 8/18 (CK8/18) and for basal cytokeratin 14 (CK14). Interestingly, Py2T cells were double-positive for these markers, while, consistent with the loss of the epithelial phenotype, Py2T LT cells only weakly stained for CK8/18 and lacked CK14 (Figure 1G *right,* see also Figure 2B).

We also performed gene expression profiling by Affymetrix DNA oligonucleotide microarray analysis of Py2T and Py2T LT cells (Table S1). The gene expression profiles were compared to molecular breast cancer subtypes using the PAM50 predictor established by Parker and colleagues [20], followed by the 9-cell line claudin-low predictor [32]. This bioinformatic analysis revealed that the gene expression profile of Py2T cells resembles a Her2-enriched breast cancer subtype, whereas the Py2T LT cell line represents the highly invasive claudin-low subtype (data not shown).

### EMT Kinetics and Plasticity in Py2T Cells

To characterize the transition from an epithelial to a mesenchymal phenotype in a time-resolved fashion, we analyzed various hallmarks of EMT upon TGFβ treatment of Py2T cells over time. On a morphological level, TGFβ treatment led to a gradual loss of cell-cell contacts and scattering already after 1 day of TGFβ treatment, while cell elongation and filopodia formation gradually increased over several days (Figure 2A). Immunoblotting analysis revealed a downregulation of E-cadherin expression over seven days, whereas N-cadherin levels began to increase between four and seven days, illustrating a classical cadherin switch (Figure 2B) [29]. Maximum fibronectin expression was observed already after one day of TGFβ treatment. Expression of the luminal CK8/18 was found reduced yet with significant expression remaining even after thirteen days of treatment, whereas the expression of basal CK14 was completely lost after seven days. We further examined the transcriptional regulation of well-known EMT markers by quantitative RT-PCR (Figure 2C). The kinetics of mRNA levels of E-cadherin, N-cadherin and fibronectin closely correlated with the immunoblotting analysis (Figure 2B and C). Furthermore, we observed a strong and gradual increase in mRNA levels of vimentin and the E-cadherin gene repressors Zeb1 and Zeb2, a robust early induction of Snail mRNA, and only a modest increase in mRNA levels of the other E-cadherin repressors Slug, E47 and Twist (Figure 2C). Overall, these time-course experiments demonstrated that in Py2T cells TGFβ-induced EMT involved gradual changes in gene expression, with early events occurring already after one day (loss of cell-cell contact, upregulation of fibronectin and Snail), while others were observed at later stages of EMT (cadherin switch, expression of vimentin, Zeb1 and Zeb2).

After having studied the transition from an epithelial to a mesenchymal state, we wondered whether Py2T cells that had undergone EMT could also revert back to the epithelial state and undergo a mesenchymal to epithelial transition (MET) upon withdrawal of TGFβ. We observed that Py2T cells cultured for up to 30 days in growth medium containing TGFβ were still able to revert to the original epithelial morphology when TGFβ was withdrawn from the medium. The MET process took approximately 18 days (Figure 2D), with a gradual re-establishment of E-cadherin expression during this time (Figure 2E). These results indicate that Py2T cells offer a valuable experimental system to study the multiple stages of EMT and its reversion, MET.

### Non-canonical TGFβ Signaling is Responsible for Early Morphological Changes

**Figure 3:**
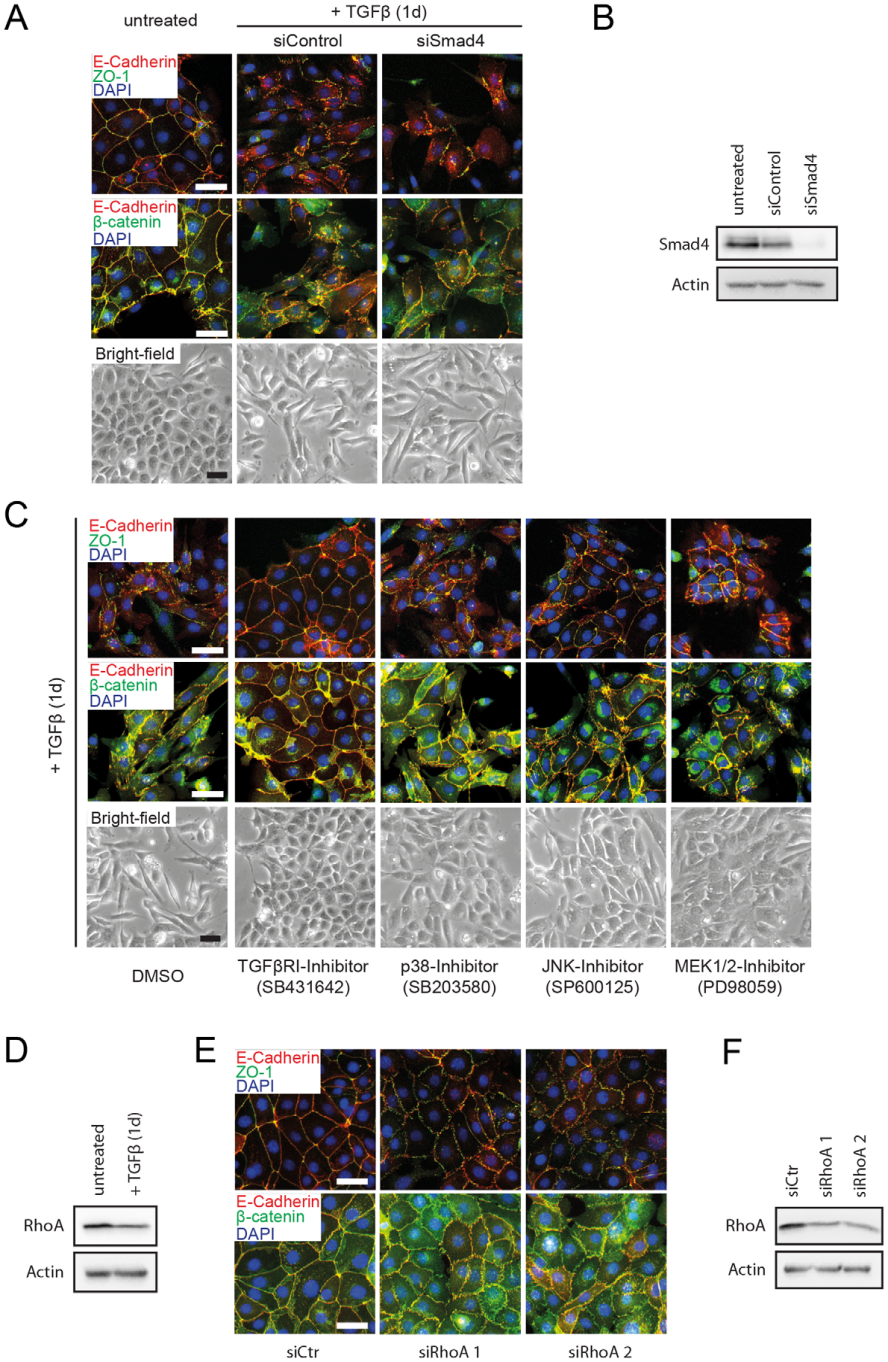
Early morphological changes and junction disassembly can be attributed to non-canonical TGFβ signaling pathways. (**A**) Smad-mediated canonical TGFβ signaling is dispensable for early changes in morphology and junction disruption. Cells were transfected with a pool of siRNAs against Smad4 or a non-targeting pool and were then treated or not with TGFβ for 1 day as indicated. Fixed cells were stained for the adherens junction components E-cadherin and β-catenin, or for E-cadherin and the tight junction component ZO-1. Note the relocalization of β-catenin from adherens junctions to the cytoplasm upon TGFβ-treatment. Scale bars, 50 µm. (**B**) Immunoblot analysis of lysates from the experiment described in (A) to control for Smad4 knockdown efficiency. (**C**) Requirement of non-canonical TGFβ signaling pathways on early morphological changes and junction disassembly. Cells were pre-treated for 4 hours with chemical inhibitors of the kinases indicated, and were then treated with TGFβ for 1 day and analyzed as described in (A). Scale bars, 50 µm. (**D**) RhoA expression levels during the early stages of EMT. Cells were treated or not with TGFβ for 1 day and RhoA expression levels were analysed by immunoblotting. (**E**) Importance of RhoA levels for tight- and adherens junction integrity. Epithelial Py2T cells were separately transfected with two different siRNAs targeting RhoA to achieve expression levels comparable to those observed in Py2T cells treated with TGFβ (see D). Cells were stained for the adherens junction components E-cadherin and β-catenin, or for E-cadherin and the tight junction component ZO-1. (**F**) Immunoblotting analysis to determine the RhoA knockdown efficiency in the experiment described in (E).

To obtain a more detailed picture of the mechanisms leading to the striking morphological alterations after the first day of EMT induction, we investigated the contribution of canonical and non-canonical TGFβ signaling to these processes. We first ablated Smad4 expression to block canonical TGFβ signaling [33]. We could not observe a block of morphological alterations or junction dissolution in cells depleted of Smad4 after one day of TGFβ treatment, indicating that canonical TGFβ signaling is not required for the initial changes in cell morphology (Figure 3A–B).

Non-canonical signaling by TGFβ involves the activation of p38 and Jnk MAP kinases via activation of Tak1 by receptor-associated TRAF6 and of Erk1/2 MAP kinase by recruitment and phosphorylation of Shc by TGFβRI and subsequent activation of MEK1/2 [34]. These mediators have been well established to contribute to TGFβ-induced EMT [35]–[40]. Indeed, inhibition of these pathways by chemical inhibitors was sufficient to at least partially block the pronounced morphological changes observed after one day of TGFβ treatment (Figure 3C). In addition, other non-canonical TGFβ-induced signals are known to contribute to EMT, such as RhoA degradation at cell junctions, which results in junction disassembly [41]. We indeed observed a slight decrease in total RhoA expression levels after one day of TGFβ treatment (Figure 3D). We experimentally mimicked TGFβ-induced downregulation of RhoA by siRNA-mediated knockdown in epithelial Py2T cells, which resulted in a partial disruption of tight and adherens junction (Figure 3E–F).

Together, these results illustrate that short-term TGFβ treatment of Py2T cells evokes cell-cell junction disassembly and pronounced phenotypic changes mainly by non-canonical TGFβ signaling.

### Migratory and Invasive Properties upon EMT Induction

**Figure 4:**
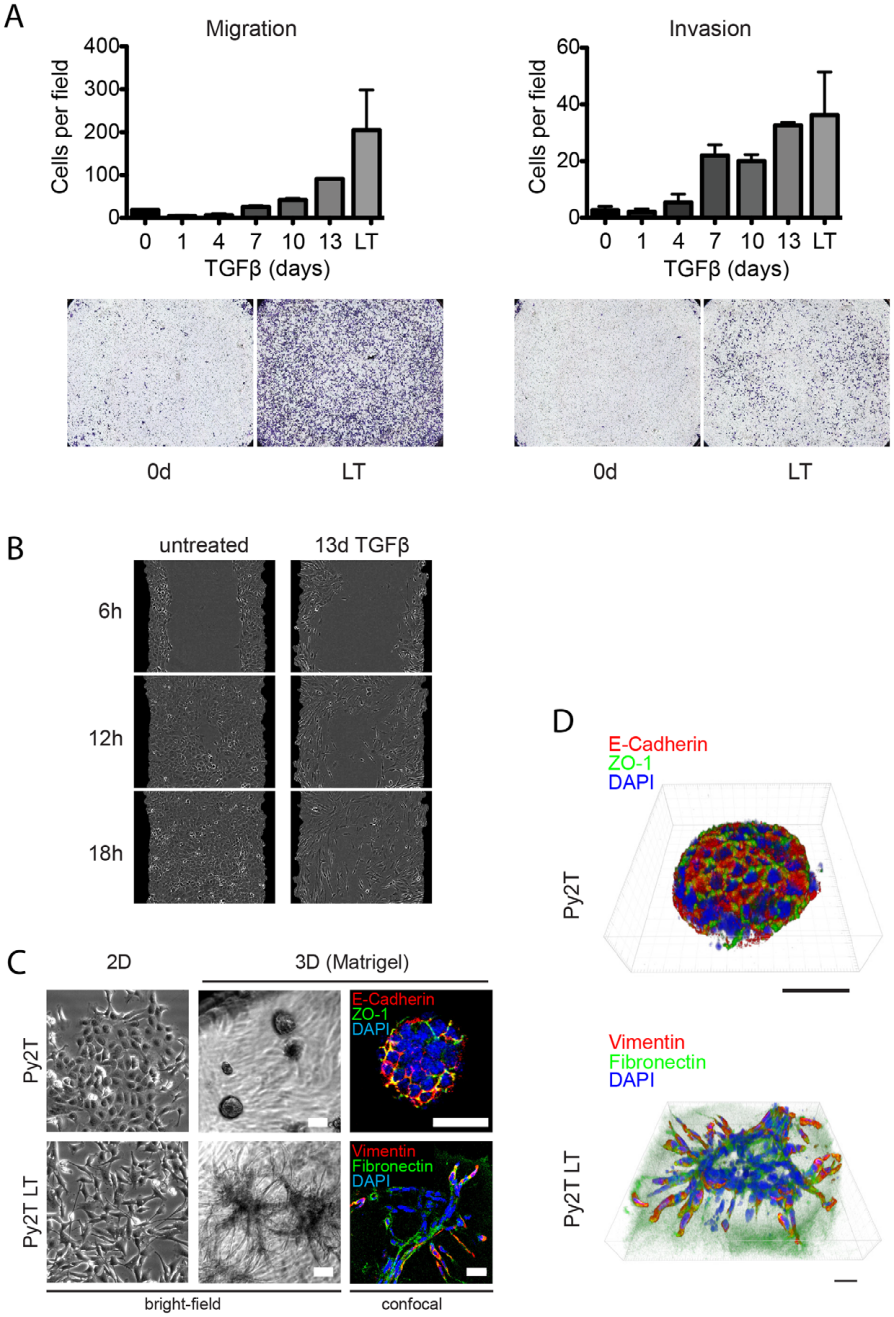
Changes of migratory and invasive properties of Py2T cells before, during and after TGFβ-induced EMT. (**A**) Boyden chamber migration and invasion assay. Cells were treated with TGFβ for the indicated times (LT = long term treatment, as described in Fig. 1F). 25'000 cells were seeded into migration or invasion chambers in duplicate in the absence or presence of TGFβ and allowed to pass through the membrane pores for 24 hours along an FBS gradient. Invasion chambers were pre-coated with growth-factor reduced Matrigel (BD BioCoat chambers). Cells that passed through the membrane pores were stained with crystal violet and photographed (*bottom panels*) and then counted (*top graphs*). Results are expressed as mean ± S.E.M of three independent experiments. (**B**) Scratch wound healing assay. Cells pre-treated with TGFβ or not as indicated were starved over night and scratch wounds were introduced into confluent monolayers. Scratch wound closure was monitored by an IncuCyte™ live cell imaging system. Black masking represents initial gap width at 0 hours. Note the collective, sheet-like wound closure by untreated Py2T cells in contrast to single cell wound infiltration of TGFβ-treated cells (also see Movies S1 and S2 for live imaging data of this experiment). (**C**) Morphology of epithelial Py2T cells and mesenchymal Py2T LT cells grown on plastic tissue culture dishes (2D) and in Matrigel (4 mg/ml; 3D). Structures were grown for 6 days, and stained directly in Matrigel with antibodies against epithelial E-cadherin and ZO-1 or against mesenchymal vimentin and fibronectin. Immunofluorescence images were acquired by confocal microscopy. Scale bars, 25 µm. (**D**) Three-dimensional reconstruction of confocal imaging stacks from cells grown in Matrigel as described in (A) (See also Movies S5 and S6 for rotating 3D models). Scale bars, 25 µm.

To evaluate whether Py2T cells could be a suitable *in vitro* model system to study functional consequences of EMT, we assessed the migratory and invasive capabilities of these cells before, during and after EMT. First, we employed a modified Boyden chamber assay to analyze whether and to what extent Py2T cells become migratory and invasive during EMT. Cells previously treated with TGFβ for different times were seeded into Boyden chamber inserts without (migration assay) or with Matrigel coating (invasion assay) and were allowed to move towards a gradient of fetal bovine serum (FBS). Quantification of cells that traversed the membrane revealed that cells treated with TGFβ for seven or more days were more migratory compared to untreated cells, and the migratory capacity dramatically increased with longer TGFβ treatment (Figure 4A *top left*). Similarly, when seeded into Boyden chambers pre-coated with Matrigel, the cells passed through the bottom of the chambers with a similar increase over the time of TGFβ treatment (Figure 4A *top right*). To illustrate these results, we stained cells located on the bottom side of the insert membranes with crystal violet (Figure 4A *bottom*). These findings clearly demonstrate that Py2T cells display a dramatic increase in chemotactic, single cell migration and invasion upon induction of EMT.

Scratch wound closure is another frequently used assay to assess the migratory capacity of cells on tissue culture plastic. Untreated and TGFβ-treated Py2T cells were grown to confluence and then starved in serum-free medium. After scratching a gap into confluent monolayers, we followed gap closure by live cell imaging (Movies S1 and S2). Figure 4B shows images at different time points after wounding. Interestingly, untreated Py2T cells closed the scratch wound already after 12 hours in a sheet-like fashion, demonstrating that they are capable of a collective mode of migration, indicative of a metastable state [42]. Py2T cells treated with TGFβ closed the scratch wound much slower, moving in a mesenchymal mode of single cell migration and displaying front-rear polarity. These observations indicate that TGFβ treatment switches Py2T cells from a collective to a single cell migration mode [43].

To compare the migratory and invasive capabilities of Py2T and Py2T LT cells in a more physiological setting, the cells were seeded into a three-dimensional extracellular matrix (Matrigel; Figure 4C). Cells cultured on plastic are shown for comparison (Figure 4C, *left*; see also Movies S3 and S4 for live imaging). When cultured for 6 days in growth factor-reduced Matrigel, Py2T cells formed spheres. In contrast, Py2T LT cells invaded the surrounding matrix (Figure 4C *middle*). To further examine these different phenotypes, we performed in-gel immunofluorescence staining of intact three-dimensional structures, followed by confocal microscopy. Double-staining of Py2T spheres with antibodies against E-cadherin and ZO-1 revealed densely packed cells with intact adherens and tight junctions, respectively (Figure 4C *top right)*. In contrast, Py2T LT structures, stained against vimentin and fibronectin, invaded the matrix as single cells or as cell trails (Figure 4C *bottom right*). The phenotypic differences between Py2T and Py2T LT cells grown in extracellular matrix became even more apparent upon reconstructing the confocal microscopy stacks to three-dimensional models using Imaris software (Figure 4D, see also Movies S5 and S6 for animation). This analysis revealed the invasion and indian-file-like trailing of Py2T LT cells as single cells. Interestingly, only the leading cells expressed vimentin, while all Py2T LT cells cultured on a two-dimensional surface were positive for vimentin (Figure 1G) and moved as single cells rather than being organized in trails (Movie S6). Taken together, these data demonstrate that the Py2T cell line represents a valuable model system to study various aspects of cell migration and invasion in the context of EMT.

### Invasive Tumor Formation upon Orthotopic Transplantation into Syngeneic Mice

**Figure 5:**
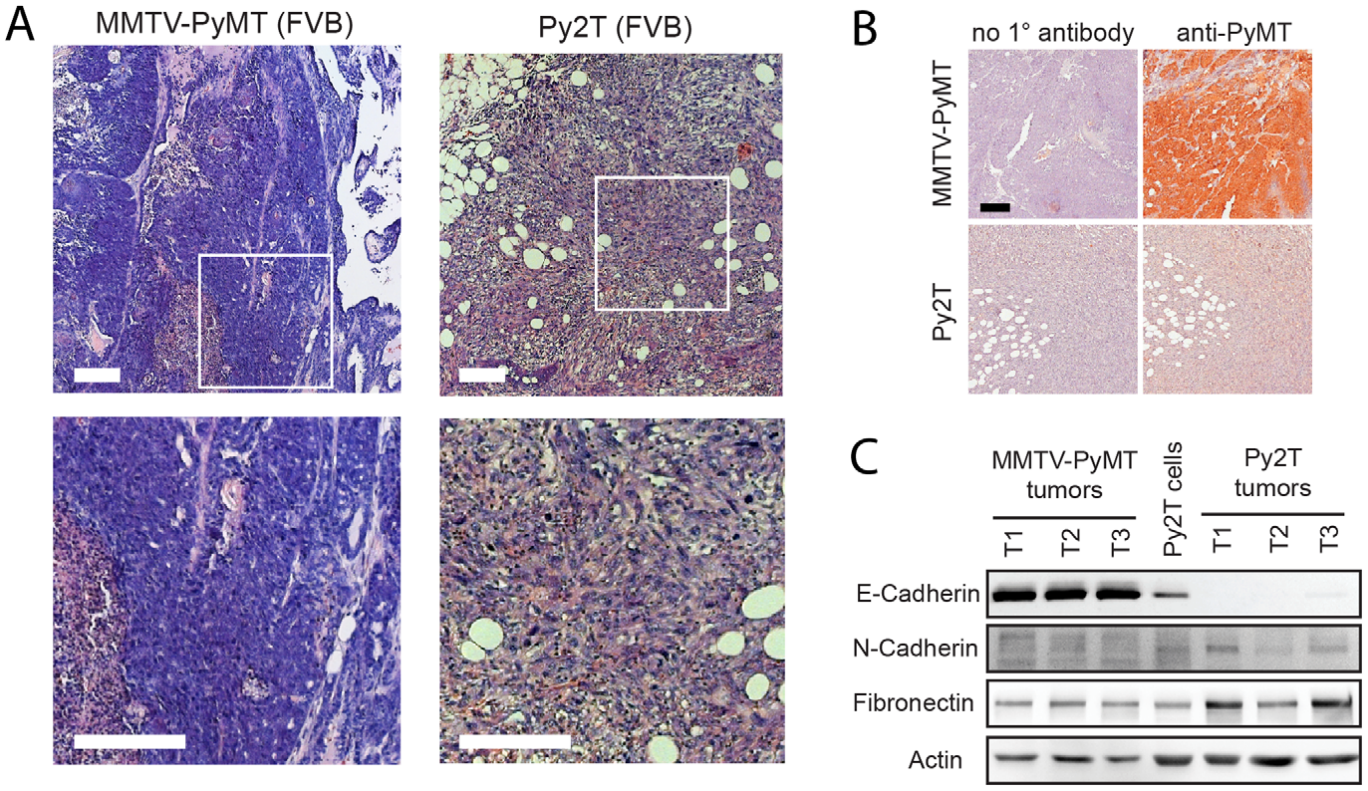
Orthotopic transplantation of Py2T cells into syngeneic mice results in the formation of invasive tumors. (**A**) H&E staining of histological sections from tumors of MMTV-PyMT transgenic mice and from transplanted Py2T tumors. 1×10^6^ Py2T cells were transplanted into the fat pad of 8 weeks old female FVB/N mice and allowed to grow tumors for 27 days. Late-stage MMTV-PyMT tumors were from 12 weeks old female mice. *Bottom panels*: enlarged regions indicated by the white squares in the top panels. Note the typical pushing borders in MMTV-PyMT tumors in contrast to stream-like invasion of fat tissue in Py2T tumors. Scale bars, 200 µm. (**B**) Polyoma-middle-T (PyMT) expression in MMTV-PyMT and Py2T tumors. Paraffin sections were stained with an antibody against PyMT. Immunohistochemical staining in the absence of primary antibody (1°) was used as negative control. Scale bar, 100 µm. (**C**) Immunoblotting analysis for EMT markers in tumor lysates of MMTV-PyMT and Py2T tumors. Lysate from cultured Py2T cells is included as a control. Note the loss of E-cadherin expression and upregulation of mesenchymal markers (N-cadherin, fibronectin) in Py2T tumors.

We next orthotopically transplanted Py2T cells into mammary fat pads of mice to evaluate their tumorigenicity. Since Py2T cells have been derived from tumors of MMTV-PyMT mice in an FVB/N background and because the PyMT transgene was no more expressed in cultured cells, we transplanted Py2T cells into syngeneic FVB/N mice. Three mice were injected with 1×10^6^ cells, all of which developed tumors. After 27 days of growth, tumors were harvested and analyzed. Haematoxylin & Eosin (H&E) staining of histological sections of a Py2T tumor (Figure 5A, *right*) and a late stage MMTV-PyMT tumor (Figure 5A, *left*) revealed that MMTV-PyMT tumors were mainly well differentiated with some less well-differentiated areas and necrosis towards the tumor center. The tumor borders were passively invading the fat pad by proliferation (pushing borders). In contrast, Py2T tumors were characterized by streams of elongated cells that were actively invading the surrounding fat tissue. Of note, Py2T tumors lacked excessive necrosis, possibly because they were well vascularized as determined by staining for the blood vessel marker CD31 (data not shown). Furthermore, Py2T tumors contained a high stromal component intermixed with tumor cells. To exclude the possibility that immune cell infiltration was due to a possible re-expression of the PyMT transgene, tumor tissue sections were stained with an antibody against the PyMT protein. As expected, PyMT expression could be detected in MMTV-PyMT tumors (Figure 5B, *left*), but not in Py2T tumors (Figure 5B, *right*). When Py2T cells were orthotopically implanted into immuno-deficient nude mice, all mice developed tumors with a substantial infiltration of CD45-positive stromal cells, with a high content of macrophages (Figure S2).

The spindle-like appearance of cells in the Py2T tumors suggested that Py2T cells might have undergone an EMT in these tumors. We thus compared lysates from mainly epithelial MMTV-PyMT tumors with lysates from mainly invasive Py2T tumors for expression of EMT markers. Indeed, expression of E-cadherin in MMTV-PyMT tumors was readily detectable as expected, however, very little if any E-cadherin expression was detectable in lysates of Py2T tumors (Figure 5C), supporting the hypothesis that Py2T cells had undergone EMT-like changes *in vivo*. Expression of the mesenchymal markers fibronectin and N-cadherin was also higher in some but not all Py2T tumors as compared to MMTV-PyMT tumors. Collectively, these results demonstrate that Py2T cells are tumorigenic, despite the absence of PyMT expression, and that they undergo oncogenic EMT-like changes *in vivo*. Notably, neither FVB/N nor immuno-deficient mice bearing Py2T tumors developed apparent metastasis, as determined by histological sectioning of various organs (data not shown).

### TGFβ-dependent EMT of Py2T Tumors

**Figure 6:**
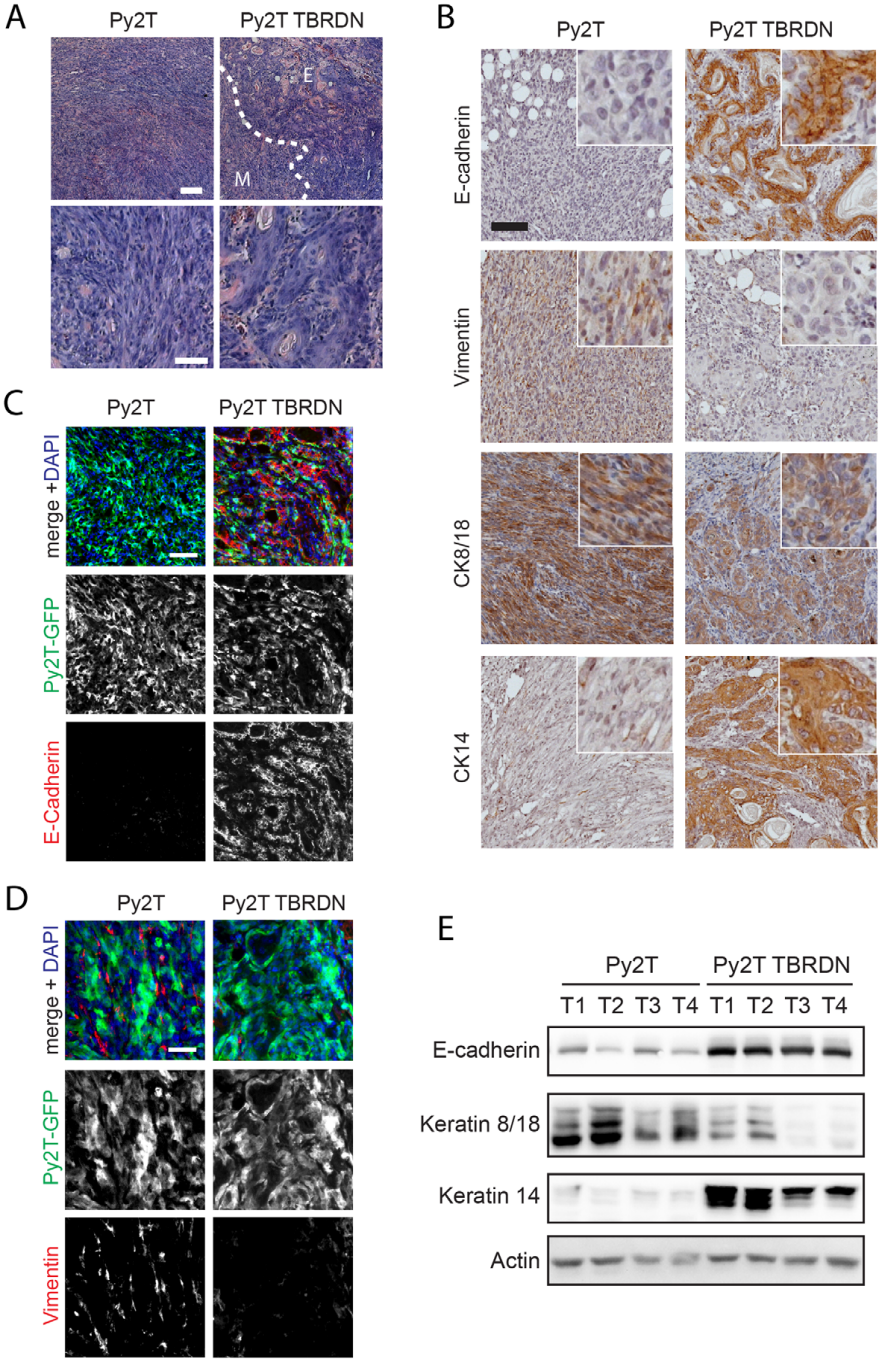
Tumors of TGFβ-resistant Py2T cells contain areas with a more epithelial phenotype. (**A**) Morphology of tumors generated from Py2T cells stably overexpressing a dominant-negative TGFβRII (Py2T TBRDN) or empty vector control cells (Py2T). 1×10^6^ cells were injected into fat pads of nude mice and tumors were grown for 24 days. Paraffin sections were stained with H&E. Note the appearance of more differentiated epithelial areas in Py2T TBRDN tumors. *Top*: Epithelial (E) and mesenchymal (M) regions are separated by the dashed line (Scale bar, 200 µm). Bottom panels show larger magnification (Scale bar, 50 µm). (**B**) Expression of EMT and lineage markers in Py2T tumors and in the more epithelial areas of Py2T TBRDN tumors. Immunohistochemical staining of paraffin sections was performed using the specified antibodies. White squares show higher magnification. Scale bar, 100 µm. (**C**) Immunofluorescence staining of frozen sections of GFP-labeled Py2T and Py2T TBRDN tumors as described in (A) with antibodies against E-cadherin *(red)* and Py2T tumor cells *(green)*. Scale bar, 20 µm. (**D**) Immunoblotting analysis of epithelial and cytokeratin lineage markers in a series of Py2T and Py2T TBRDN tumors as indicated. (**E**) Immunofluorescence staining of frozen sections of GFP-labeled Py2T and Py2T TBRDN tumors as described in (A) with antibodies against vimentin *(red)* and Py2T tumor cells *(green)*. Scale bar, 20 µm.

We next assessed whether the EMT occurring during Py2T tumor growth in the mammary fat pad of mice could be attributed to stimulation by host-derived TGFβ. First, we generated Py2T cell lines that stably express GFP for their distinction from host stromal cells. Next, we superinfected these cells with a lentiviral construct encoding a dominant-negative form of TGFβ receptor II (TBRDN) [26] or empty vector as control. Cultured Py2T TBRDN-expressing cells did not show any apparent changes in phenotype as compared to control cells in the absence of TGFβ, but were resistant against TGFβ-induced EMT (Figure S3A). In a next step, we transplanted Py2T control and Py2T TBRDN into fat pads of immuno-deficient nude mice to evaluate their ability to undergo EMT *in vivo.* All mice developed tumors, and tumor growth was not significantly different between the two experimental groups, although TBRDN tumors tended to grow more slowly with increasing size in comparison to Py2T control tumors (Figure S3B). H&E staining of Py2T control tumors revealed the same stream-like cellular growth pattern as observed in Py2T tumors in FVB/N mice (Figure 6A *top left*), with cells displaying a spindle-like morphology (Figure 6A *bottom left*). Interestingly, tumors formed by Py2T TBRDN contained patches of more differentiated appearance (Figure 6A *top right*), with cells adopting a round, differentiated morphology (Figure 6A *bottom right*). However, Py2T TBRDN tumors also contained a significant portion of mesenchymal areas (Figure 6A *right*), suggesting that in these areas, Py2T cells underwent EMT in response to signals other than TGFβ.

Analysis of the expression of EMT markers revealed that, Py2T control tumors were negative for E-cadherin expression (Figure 6B, *top left*), whereas the more differentiated regions in TBRDN tumors strongly expressed E-cadherin (Figure 6B, *top right*). These results indicated that the inhibition of TGFβ signaling in Py2T TBRDN cells was sufficient to prevent a loss of E-cadherin expression and to preserve an epithelial phenotype in some but not all tumor areas. Immunofluorescence microscopy analysis of E-cadherin staining of GFP-expressing Py2T and Py2T TBRDN tumor cells, respectively, confirmed these observations (Figure 6C). Furthermore, immunoblotting analysis demonstrated higher E-cadherin expression in Py2T TBRDN tumors in contrast to Py2T control tumors (Figure 6D). Py2T tumors also contained a large amount of cells that stained positive for the mesenchymal marker vimentin, however, these vimentin expressing cells represented stromal cells rather than Py2T cells, as revealed by a lack of GFP expression (Figure 6E). Hence, although capable of vimentin upregulation upon EMT induction *in vitro* (Figure 2C), Py2T cells failed to upregulate vimentin *in vivo*, suggesting that EMT in transplanted tumors is incomplete, which is often reported as a hallmark of oncogenic EMT [44]; (see also Discussion).

As Py2T cells expressed both luminal (CK8/18) and basal (CK14) markers in culture (Figure 1G, Figure 2B), we were curious to see whether the EMT-like changes observed in tumors would be accompanied by changes in the expression of these cell lineage markers. Immunohistochemistry staining (Figure 6B) and immunoblotting analysis (Figure 6D) revealed a switch-like change in expression: a loss of CK14 expression was observed in favor of CK8/18 expression in Py2T tumors. On the other hand, the epithelial patches of Py2T TBRDN tumors were strongly positive for CK14 expression and displayed a reduction or even a loss of CK8/18 expression. Together, these results demonstrate that Py2T tumors display EMT-like changes characterized by a loss of E-cadherin expression, and suggest an apparent differentiation along the luminal lineage, both of which is inhibited in distinct tumor areas by blocking the TGFβ responsiveness of the tumor cells.

## Discussion

We herein report the generation and characterization of a stable murine breast cancer cell line, named Py2T, from a primary breast tumor of an MMTV-PyMT transgenic mouse. Cultured Py2T cells can be induced to undergo a full EMT by TGFβ treatment, a multistage process that takes up to ten days and results in a complete loss of epithelial morphology and epithelial marker expression, and the gain of mesenchymal marker expression and increased cell migration and invasion. Upon long-term treatment with TGFβ, Py2T cells maintain the mesenchymal differentiation status (Py2T LT), allowing the direct comparison between the extreme stages of epithelial-mesenchymal plasticity. Upon removal of TGFβ, Py2T LT cells revert to their epithelial origin by undergoing an MET, with the gain of epithelial morphology and marker expression. Our pharmacological interference studies reveal that the early stages of TGFβ-induced EMT in Py2T cells depend on non-canonical TGFβ signaling, involving Jnk, p38 and ERK1/2 MAP kinase signaling and the degradation of the small GTPase RhoA. In contrast, Smad4 and with it canonical TGFβ signaling appears to be dispensable for this process.

Py2T cells also offer a novel syngeneic orthotopic transplantation model of malignant breast cancer progression. Upon injection into the fat pads of syngeneic FVB/N mice or into immuno-deficient nude mice, Py2T cells form primary tumors and spontaneously undergo EMT-like changes *in vivo*. As a proof of concept for the dual *in vitro* and *in vivo* use of Py2T cells as models of murine breast cancer cells undergoing EMT, we blocked TGFβ responsiveness of Py2T cells by stable expression of a dominant-negative version of TGFβRII. Transplantation of these cells yielded tumors containing areas with an epithelial phenotype, showing that the EMT-like changes in Py2T cell-derived tumors are, at least in part, dependent on TGFβ stimulation. These experiments approve Py2T cells as a versatile model for functional studies of murine breast cancer cells undergoing EMT *in vitro* and *in vivo*.

It has been recognized that breast cancer is not a single, but a heterogenous disease of various subtypes, which can be categorized according to staining for marker combinations, or, more recently, by molecular subtyping according to gene expression profiles. The type of breast cancer is largely dictated by the transforming oncogene and the cell of origin being transformed [45]–[48]. We therefore characterized the cell type represented by Py2T cells. Molecular subtyping of MMTV-PyMT tumors has previously shown that these tumors resemble the luminal subtype of human breast cancer [45], [49], as would be expected from the fact that the MMTV promoter is active in luminal epithelial cells [50], [51]. Consistent with their origin from a tumor of an MMTV-PyMT transgenic mouse, Py2T cells are positive for the luminal markers estrogen receptor (ER) and CK8/18 (Figure 1G). Interestingly, Py2T cells also co-express the basal marker CK14 (Figure 1G) and therefore do not display a purely luminal phenotype. Concomitant basal and luminal cytokeratin expression has also been observed in a luminal breast cancer model where the MMTV promoter has been used to drive mutant PIK3CA H1047R oncogene expression [52], and one of the pathways activated by PyMT is the PI3K pathway [53], suggesting that similar mechanisms are involved. Our observations and those of others show that MMTV-PyMT tumors also contain a fraction of CK14-positive tumor cells (data not shown) [54]. Furthermore, simultaneous expression of CK8/18 and CK14 has been established as a hallmark of basal cell lines [55].

Together, these considerations suggest that Py2T cells should be categorized as a basal cell line with luminal origin. It is interesting to note in this context that EMT-like changes have most commonly been observed in the basal-like subgroup of breast cancers, indicating that this subgroup is predisposed for EMT-like changes [56], [57]. Basal-like tumors also encompass the recently determined claudin-low subtype, now considered to be a distinct entity, which is clearly enriched in EMT marker expression [21], [58], [59]. Our gene expression profiling and subsequent bioinformatic analysis according to the PAM50 and 9-cell line claudin-low predictor [20], [32] revealed that Py2T cells most closely resemble Her2-enriched breast cancer of patients. In contrast, Py2T cells that have undergone TGFβ-induced EMT (Py2T LT) resemble basal-like, claudin-low breast cancer, a highly invasive breast cancer subtype that has been shown to correlate with EMT in a variety of experimental systems [32], [58], [60], [61].

Expression of basal cytokeratins 5 and 14 has also been linked to a hybrid or metastable differentiation state, in which cells display considerably more plasticity than fully differentiated cells, residing in a dynamic continuum between epithelial and mesenchymal states [42], [44]. One feature that characterizes metastable cells is that they display loose but intact cell-cell adhesions and show migratory properties in the form of collective movement as a sheet. Indeed, when grown to confluence, Py2T cells close a scratch wound as a cellular sheet (Figure 4B and Movie S1). A further indicator for a metastable state is the observation that, when grown under sparse culture conditions on plastic, Py2T cells are able to transiently leave the epithelial sheet and move as single cells in a spontaneous manner (Movie S3). This single cell mode of migration resembles amoeboid movement, characterized by a rounding of cell bodies and a fast change in direction, and is distinct from the mesenchymal mode of migration characterized by front-rear polarity which we observed with Py2T LT cells (Movie S4) [43], [62]. The reversibility of TGFβ-induced EMT of Py2T cells further illustrates the plasticity of Py2T cells and has also been proposed as a hallmark of metastability (Figure 2E) [42], [44], [63]. From these observations we conclude that cultured Py2T cells do not represent fully differentiated epithelial cells, but that they are rather in a metastable state that is readily shifted towards a mesenchymal phenotype by TGFβ treatment.

When implanted into the mammary fat pad microenvironment, Py2T cells eventually develop tumors with an EMT-like phenotype (Figures 5 and 6). We believe that the term „EMT-like“ is accurate, since we have noticed that in these tumors, Py2T cells do not completely convert into mesenchymal cells as they do under culture conditions in the presence of TGFβ. Breast cancers can display a range of stages of EMT, in fact, tumor-associated EMT appears less complete than developmental EMT [7], [64]. A staging scheme has been proposed based on the state of cell polarization, cell cohesiveness and intermediate filament expression, categorizing oncogenic EMT into four distinct stages (P0–P3), with P0 designating full epithelial differentiation and P3 indicating a fully mesenchymal state [44]. Py2T tumors correspond to the P2 stage, where cells have lost polarization and cohesive cell-cell contacts, but retain cytokeratin expression (at least CK8/18) and fail to upregulate vimentin (Figure 6). When we block TGFβ-responsiveness in Py2T cells, epithelial morphology is retained in distinct areas, where tumor cells appear to be organized as dynamic cohesive sheets or strand-like structures, however not regaining full epithelial polarization (Figure 6). This phenotype is again consistent with a metastable state rather than full epithelial differentiation, and corresponds to the P1 stage of oncogenic EMT according to [44].

Despite the fact that Py2T cells form locally invasive tumors and that MMTV-PyMT tumors give rise to distant metastases, we were unable to detect any apparent metastases evoked by Py2T tumors. One conceivable reason for this apparent discrepancy could be the following: Py2T tumors appeared fast growing and aggressive and, due to animal welfare considerations, mice had to be sacrificed approximately 25 days after implantation (Figure S3B). Therefore, the timeframe to establish detectable metastasis may be simply too short. In comparison, the metastasis latency in MMTV-PyMT tumors is about 3.5 months [65].

We have observed that PyMT transgene expression is absent in Py2T cells both *in vitro* (Figure 1C) and *in vivo* (Figure 5B). This finding has important implications. First, it allows the transplantation of Py2T cells (derived from MMTV-PyMT mice in a FVB/N background) into syngeneic FVB/N mice (Figure 5). Second, the loss of PyMT expression together with the fact that these cells are nevertheless tumorigenic suggests that outgrowing Py2T cells that have undergone EMT have escaped oncogene addiction. In support of this hypothesis, we observed that mesenchymal Py2T LT cells formed significantly more colonies when grown under anchorage-independent conditions in soft agar (Figure S4). Intriguingly, in several other mouse models of breast cancer, discontinued oncogene expression is followed by the appearance of tumors that display EMT-like features (see reference [66] for review). For example, after turning off Her2/neu expression in tumors induced by this oncogene in the mammary gland, tumors regress, yet regrow as spindle cell „EMT“ tumors that are strikingly similar if not identical in phenotype to the tumors we describe here [67]. In agreement with our study, these tumors have not been observed to metastasize [66]. It is likely that our model recapitulates these events, whose underlying mechanisms have yet to be determined. If so, the Py2T model system could be instrumental in elucidating mechanisms of tumor recurrence and of therapy resistance development, which has been previously attributed to EMT [13], [15], [64], [68], [69]. Finally, in light of the recent findings that EMT confers stem cell-like traits to cancer cells [10], [11], Py2T cells also offer a unique system to study these events *in vitro* and *in vivo*.

### Conclusions

We have established and functionally characterized a novel cellular model of murine breast cancer EMT (Py2T). While Py2T cells undergo EMT in response to TGFβ stimulation *in vitro*, orthotopic transplantation into mice results in tumors displaying oncogenic, TGFβ-dependent EMT. Py2T cells thus represent a versatile model to investigate the molecular mechanisms underlying EMT and to delineate how EMT contributes to therapy resistance, loss of oncogene addiction and tumor recurrence.

## Materials and Methods

### Antibodies and Reagents

Antibodies: PyMT (mouse monoclonal Pab762, a kind gift of Dr. S. Dilworth, Imperial College London), Actin (sc-1616, SantaCruz Biotechnology), E-cadherin (610182, Transduction Laboratories, used for immunoblotting and IHC), E-cadherin (13-1900, Zymed, used for immunofluorescence stainings), N-cadherin (M142, Takara Bio), fibronectin (F3648 Sigma-Aldrich), GAPDH (ab9485, Abcam), cytokeratin 14 (RB-9020-P0, NeoMarkers), cytokeratin 8/18 (20R-CP004, Fitzgerald), vimentin (V2258, Sigma-Aldrich), ERα (sc-542, Santa Cruz Biotechnology), ZO-1 (617300, Zymed), F4/80 (MCAP497, Serotec), CD45 (550539, BD), Smad2/3 (610842, BD), Smad3 pSer423/425 (9520, Cell Signalling), β-catenin (C2206, Sigma-Aldrich), Smad4 (sc-7966, Santa Cruz Biotechnology), RhoA (sc-418, Santa Cruz Biotechnology).

Reagents: recombinant human (rh) TGFβ1 (240-B-010, R&D Systems), recombinant mouse (rm) EGF (PMG8041, Invitrogen), rmIGF-1 (250-19, Peprotech), rmHGF (2207-HG, R&D Systems), rmbasicFGF (3139-FB-025, R&D Systems), rhPDGF-BB (220-BB, R&D Systems, rhIL-6 (200-06 Peprotech), Dexamethasone (800-437-7500, Chemicon), Matrigel, growth factor reduced (356230, BD), SB431542 (S4317, Sigma Aldrich), SB203580 (ALX-270-339, Axxora), SP600125 (ALX-270-339, Axxora), PD98059 (ALX-385-023, Axxora).

### Cells and Cell Lines

A subclone of NMuMG cells (NMuMG/E9; hereafter NMuMG) was a kind gift of Dr. M. J. Wheelock and has been previously described [17]. NMuMG cells were originally obtained from the American Type Culture Collection (ATCC, Manassas, VA). Py2T cells were isolated from a breast tumor of an MMTV-PyMT female mouse with an FVB/N background. Isolation of this cell line was done with approval, and according to the rules and guidelines of, the Swiss Federal Veterinary Office (SFVO) and the local ethics committee (Cantonal Veterinary Office, Basel-Stadt, Switzerland); (see also Ethics Statement at the end of this section). NMuMG and Py2T cells were cultured in DMEM supplemented with glutamine, penicillin, streptomycin, and 10% FBS (Sigma).

### Mouse Strains

MMTV-PyMT [18], [19] were received from N. Hynes (FMI, Basel, Switzerland). BALB/c nude mice were purchased from JANVIER SAS (Le Genest Saint Isle, France).

### Primers

Primers used for quantitative RT-PCR are listed in Table S2. For genotyping of the MMTV-PyMT transgene, the following primers were used: MMTV-PyMT (forward: 5′-cggcggagcgaggaactgagg-3′, reverse: 5′-tcagaagactcggcagtcttag-3′).

### Genotyping

To extract DNA, cells from a confluent 10 cm dish were trypsinized, washed in PBS and pelleted. To the pellet, 450 µL tail tip buffer (50 mM Tris-HCl pH 8, 100 mM NaCl, 100 mM EDTA, 1% SDS) and 180 µL 6 M NaCl were added, the samples were mixed and spun at full speed in a tabletop centrifuge. Supernatant was added to 600 µL isopropanol, vortexed and spun for 5 min at full speed. Supernatant was discarded and 500 µL of 70% EtOH was added, vortexed and spun for 3 min at full speed. Supernatant was discarded and the pellet was dried and resuspended in TE buffer. Samples were analyzed using standard PCR procedure.

### Quantitative RT-PCR

Total RNA was prepared using Tri Reagent (Sigma-Aldrich), reverse transcribed with M-MLV reverse transcriptase (Promega, Wallisellen, Switzerland), and transcripts were quantified by PCR using SYBR-green PCR MasterMix (Applied Biosystems, Rotkreuz, Switzerland). Riboprotein L19 primers were used for normalization. PCR assays were performed in triplicates, and fold induction was calculated using the comparative Ct method (ΔΔC_t_).

### Microarray Gene Expression Profiling and Expression Analysis

RNA was isolated from Py2T and Py2T LT cells using QIAzol (Quiagen). RNA quality and quantity was evaluated using an Agilent 2100 Bioanalyzer (Agilent Technologies). The manufacturer’s protocols for the GeneChip platform by Affymetrix were followed. Methods included synthesis of the first- and second-strand cDNA followed by synthesis of cRNA by in vitro transcription, subsequent synthesis of single-stranded cDNA, biotin labeling and fragmentation of cDNA and hybridization with the microarray slide (GeneChip® Mouse Gene 1.0 ST array), posthybridization washings and detection of the hybridized cDNAs using a streptavidin-coupled fluorescent dye. Hybridized Affymetrix GeneChips were scanned using an Affymetrix GeneChip 3000 scanner. Image generation and feature extraction were performed using Affymetrix GCOS Software and quality control was performed using Affymetrix Expression Console Software. All microarray raw data has been uploaded to the ArrayExpress Database (Accession number E-MEXP-3731 and is publicly available (www.ebi.ac.uk/arrayexpress/).

Microarray data was analysed using R statistical programming (R2.13.0; www.r-project.org) and its Bioconductor packages (http://www.bioconductor.org). Gene expression was calculated after RMA normalization and linear modeling using the limma package. The probesets were annotated to mouse Refseq IDs with the brainarray annotation package (http://brainarray.mbni.med.umich.edu) and human homologues were mapped using biomart (http://www.biomart.org/). Differentially expressed genes were determined with Empirical Bayes Statistics according to the following criteria: expression change between Py2T and Py2T LT of at least 2 fold, an average log expression of at least 3 and logOdds of at least 0.

### Molecular Subtyping

First, intrinsic subtype classification into Luminal A, Luminal B, Basal-like, HER2-enriched and Normal-like groups was performed using the 50 gene (PAM50) predictor, comparing Py2T and Py2T LT to the UNC337 training set provided by Parker et al. [20]. Briefly, the centroids from 50 intrinsic genes were compared between the training set and the cell lines analysed here using Spearman’s rank correlation to predict the subtype on the test set using PAM50 predictor bioclassifier R script with R2.13.0 (www.r-project.org) [20]. In a second step, Claudin-low subtype prediction was performed as described by Prat et al. [21]. Briefly, centroids for “claudin-low” or “others” were calculated on the training set provided by Prat et al. [21] including different breast cancer cell lines from the Neve et al. study [22]. For each novel cell line to be classified, the Euclidean distance to the centroids from the training set was calculated and the subtype assigned according to the nearest centroid. Classification was performed using R2.13.0 (www.r-project.org).

### Luciferase Reporter Assay

5×10^4^ Py2T cells were plated in triplicate in a 24 well-plate. One day after plating, cells were transfected with 800 ng reporter and 5 ng Renilla encoding plasmids using Lipofectamine 2000. Fresh growth medium was added after 5 hours of transfection containing 2 ng/mL TGFβ or not. After 2 days, cells were lysed directly in plates using 1× passive lysis buffer (#E194, Promega) and lysates were analyzed using the Dual-Luciferase Reporter Assay System (#E1960, Promega) and a Berthold Luminometer LB960. Measured luciferase values were normalized to internal Renilla control. The Smad4 reporter was kindly provided by Dr. P. ten Dijke (Leiden University; [23].

### Cell Line Isolation

A piece (∼200 mg) of freshly isolated tumor was transferred into collection medium (DMEM supplemented with 10% FBS, 2 mM glutamine, supplemented with Gentamycin (50 ug/mL)) and minced into very small pieces using sterile technique with a scalpel. Pieces were collected by rinsing with pre-digestion buffer (10 mM HEPES pH 7.4, 142 mM NaCl, 0.67 mM KCl, 1 mM EDTA) supplemented with Gentamycin (50 µg/mL)(G1397, Sigma-Aldrich) and 1× Antibiotic-Antimycotic (15240-096, Invitrogen), and transferred to a 15 mL Falcon tube. Pieces were predigested in horizontal position at 200 rpm at 37°C for 30 min on a bacterial shaker. Predigested tissue was pelleted by spinning at 900×g for 5 min, the supernatant was removed and the pellet was resuspended in digestion mix (10 mM HEPES pH 7.4, 142 mM NaCl, 0.67 mM KCl, 0.67 mM CaCl2, 20 mM Glucose, 1 mg/mL Collagenase Type I, 0.1 mg/mL DNAseI) supplemented with Gentamycin (50 µg/mL) and 1× Antibiotic-Antimycotic. The tissue was digested by shaking in horizontal position at 200 rpm at 37°C for 30 min on a bacterial shaker. For final single cell dissociation, tissue was pipetted up and down for 5 min using a 1 mL pipette. Digested tissue was pelleted, washed twice in PBS and plated into multiple wells of a 24 well-plate in normal growth medium (DMEM supplemented with 10% FBS, 2 mM glutamine, 100 U penicillin and 0.2 mg/ml streptomycin). Growth medium was exchanged the next day, and subsequently exchanged every three to four days until epithelial cultures without Fibroblast contamination emerged.

### Immunofluorescence Staining of Cultured Cells

Cells were plated on glass coverslips and treated for the indicated times with TGFβ. The following steps were all done at room temperature. After fixation using 4% paraformaldehyde/PBS for 15 min, cells were permeabilized with 0.5% NP-40 for 5 min. Next, cells were blocked using 3% BSA, 0.01% TritonX-100 in PBS for 20 min. Then, cells were incubated with the indicated primary antibodies for 1 h followed by incubation with the fluorochrome-labeled secondary antibody (Alexa Fluor®, Invitrogen) for 30 min at room temperature. Nuclei were stained with 6-diamidino-2-phenylindole (DAPI) (Sigma-Aldrich) for 10 min. The coverslips were mounted (Fluorescent mounting medium, Dako) on microscope slides and imaged with a conventional immunofluorescence microscope (Leica DMI 4000) or a confocal microscope (Zeiss LSM 510 Meta). Confocal stacks were reconstructed with Imaris Software (Bitplane, Switzerland).

### Immunoblotting

Cells were lysed in RIPA buffer (150 mM NaCl, 2 mM MgCl, 2 mM CaCl_2_, 0.5% NaDOC, 1% NP40, 0.1% SDS, 10% Glycerol, 50 mM Tris pH 8.0) containing 2 mM Na_3_VO_4_, 10 mM NaF, 1 mM DTT, and a 1∶200 dilution of stock protease inhibitor cocktail for mammalian cells (Roche). Protein concentration was determined using the BCA assay kit (Pierce). Equal amounts of protein were diluted in SDS-PAGE loading buffer (10% glycerol, 2% SDS, 65 mM Tris, 1 mg/100 ml bromophenol blue, 1% β-mercaptoethanol) and resolved by SDS-PAGE. Proteins were transferred to polyvinylidene fluoride (PVDF) membranes (Millipore) by semi-dry transfer, blocked with 5% skim milk powder in TBS/0.05% Tween 20 and incubated with the indicated antibodies. HRP conjugated secondary antibodies were detected by chemiluminescence using a Fusion F×7 chemiluminescence reader (Vilber Lourmat, France).

### Retroviral Infection

A cDNA encoding EGFP was subcloned from pEGFP-N3 (Clontech) into the retroviral vector pBabe-hygro [24]. The resulting plasmid pBH-EGFP was transfected into the retroviral packaging cell line Plat-E (purchased from Cell Biolabs) [25] using FugeneHD (Roche). One day after transfection, medium was exchanged and retroviral supernatant was produced for 2 days. Viral supernatant was filtered through 0.45 µm pores and 8 µg/mL Polybrene was added. Py2T cells were plated into 6-well plates and were infected with viral supernatant one day after plating. For infection, 2 mL supernatant was added per well and plates were spun for 1 hour at 30°C at 1000×g and were subsequently incubated at 37°C with 5% CO_2_ in a tissue culture incubator for 2 more hours. Viral supernatant was then replaced by normal growth medium and one day later, selection with 500 µg/mL Hygromycin B (Invitrogen) was performed for 5 consecutive days.

### Lentiviral Infection

A cDNA encoding a human dominant-negative version of TGFβRII (K277R) [26] (kindly provided by M. Oft, Targenics Inc., San Francisco) was subcloned into the lentiviral expression vector pLentiCMV (a kind gift from O. Pertz, University of Basel). Lentiviral particles were produced by transfecting HEK293T cells with the lentiviral expression vector pLentiTBRDN or empty vector as a control, in combination with the helper vectors pHDM-HGPM2, pHDM-Tat1b, pRC-CMV-RaII and the envelope encoding vector pVSV using Fugene HD. After two days of virus production, lentivirus-containing supernatants were harvested, filtered (0.45 µm) and added to target cells in the presence of polybrene (8 µg/ml). Cells were spun for 1 hour at 30°C at 1000×g and were subsequently incubated at 37°C with 5% CO_2_ in a tissue culture incubator for 2 more hours. Viral supernatant was then replaced by normal growth medium and one day later, selection with 5 µg/mL Puromycin (Sigma-Aldrich) was performed for 3 consecutive days.

### Boyden Chamber Migration and Invasion Assay

Cells pre-treated or not with TGFβ were trypsinized, washed once with PBS, and resuspended in growth medium containing 0.2% FBS and 2 ng/mL TGFβ where appropriate. 2.5×10^4^ cells in 500 µL were seeded into cell culture insert chambers containing 8 µm pores (migration chambers: 353097, BD Falcon; invasion chambers with ECM coating: 354483, BD Falcon) in triplicate. Subsequently, the bottoms of chambers were filled with 700 µL of growth medium containing 20% FBS, and cells were incubated in a tissue culture incubator at 37°C with 5% CO_2_. After 24 hours, inserts were fixed with 4% PFA/PBS for 10 min. Cells that had not crossed the membrane were removed with a cotton swab, and cells on the bottom of the membrane were stained with DAPI. Images of five fields per insert were taken with a Leica DMI 4000 microscope and stained cells were counted using an ImageJ software plugin developed in-house. Subsequently, inserts were stained in crystal violet solution (0.125% crystal violet, 20% MeOH) for 10 minutes, followed by washing in a large volume of dH_2_O and drying over night. Images of crystal violet stained inserts were taken with an AxioVert microscope (Zeiss, Germany).

### Scratch Wound Closure Assay

3×10^5^ untreated Py2T cells and 3×10^5^ Py2T cells treated with TGFβ for 13 days were seeded into 24-well plates with or without TGFβ. Normal growth medium was replaced by starving medium containing 2% FBS with or without TGFβ on the next day. After starvation over night, a wound was scratched into confluent monolayers and plates were transferred to an Incucyte™ live imaging instrument (Essen BioScience).

### 3D Matrigel Culture and In-gel Immunofluorescence Staining

Growth factor-reduced Matrigel (356230, BD) stock was thawed on ice and diluted to 4 mg/mL protein with ice-cold, serum-free growth medium. Cells were trypsinized, resuspended in ice-cold normal growth medium and counted using a CASY cell counter (Roche, Switzerland). A pellet of 2500 cells was resuspended in 10 µL of pre-diluted Matrigel and transferred to one well of a µ-slide angiogenesis microscopy slide (ibidi, Martinsried, Germany). After an incubation of 20 min in a tissue culture incubator to allow solidification of the gel, 50 µL of normal growth medium containing or not 2 ng/mL TGFβ was added to each well. Growth medium was replenished every third day. After 6 days of growth, structures were prepared for immunofluorescence analysis directly in the matrix. Structures were fixed with 4% PFA/PBS for 10 min and washed with 20 mM glycine/PBS for 5 min. After a second wash with PBS, cells were permeabilized and blocked with IF buffer (0.2% TritonX-100/0.1% BSA/0.05% Tween20/PBS) containing 10% goat serum. Samples were incubated with primary antibodies diluted in IF buffer for 2 hours at room temperature in a humid chamber. After 2 washes with IF buffer, secondary antibodies diluted in IF buffer were incubated for 45 minutes, and nuclei were stained with DAPI solution for 20 minutes. After 2 final washes with IF buffer, samples were topped with fluorescent mounting medium (Dako) and imaged with a confocal microscope (LSM 510 Meta, Zeiss).

### Soft Agar Colony Formation Assay

Cells were seeded into 6-well plates at 1×10^4^ cells per well in 0.35% agarose/DMEM complete growth medium onto a base layer consisting of 0.5% agarose/DMEM complete growth medium. Growth medium containing 2 ng/mL TGFβ or not was added on top of the agarose layers, and was replaced every four days. After 10 days, viable colonies were stained with MTT solution (Sigma-Aldrich) and were counted.

### siRNA-mediated Knockdown

To achieve knockdown of Smad4, 10 nM final concentration of siGENOME smart pool siRNAs (Dharmacon, M-040687-00-0005) were used. A non-targeting pool was used as a control (Dharmacon, D-001810-10-20). Two different, custom-designed siRNAs against RhoA with the following sequences were used at 10 nM final concentration: siRhoA1 gaaggcagagauauggcaa(dT)(dT), siRhoA2 ugaagcaggagccgguaaa(dt)(dT). Negative Universal Control Medium (45-2001, Invitrogen) served as negative control. Reverse transfection of siRNAs was performed with Lipofectamine RNAiMax reagent (Invitrogen) according to the manufacturer’s instructions.

### Orthotopic Tumor Cell Transplantation

Cells were trypsinized, washed twice and resuspended in ice-cold PBS. Eight weeks old female BALB/c nude mice or FVB/N mice were anaesthetized with isoflurane/oxygen and injected with 1×10^6^ Py2T cells in 100 µL PBS into mammary gland number 9. Tumor volumes were calculated according to the formula V = 0.5*D*d∧2, where D represents length and d represents width of tumors measured by a digital caliper. Mice were sacrificed by CO_2_ and tumors were isolated and further processed.

### Histology and Immunostaining

For immunohistochemistry (IHC) and Haematoxylin & Eosin (H&E) stainings, tumors were fixed at 4°C in 4% phosphate-buffered paraformaldehyde (PFA) for 12 hours and then embedded in paraffin after ethanol/xylene dehydration. H&E staining was performed as previously described [27], [28]. For immunofluorescence analysis of frozen sections, organs were fixed at 4°C in 4% PFA for 2 hours, and cryopreserved for 10 hours in 20% sucrose in PBS prior to embedding in OCT freezing matrix. For IHC stainings of PFA-fixed, paraffin-embedded specimens, antigen epitopes were retrieved by boiling slides in 10 mM Na-Citrate buffer (pH 6.0) in a PrestigeMedical Z2300 antigen retriever. Stainings with mouse and rabbit antibodies were performed using the Dako EnVision plus Kit (K4065) according to the manufacturer’s recommendations. Cytokeratin 8/18 staining was performed using the Vectastain ABC kit (PK-6100 standard, Vector). Stainings were revealed by incubation with biotinylated secondary antibodies and ABC Elite detection kit using AEC substrate (all from Vector Laboratories) according to the manufacturer’s instructions and counterstained using hematoxilin. Cryosections were cut 7 µm thick and dried for 30′ prior to rehydration in PBS. Slides were permeabilized with in PBS/0.2% TritonX-100 and blocked for 30 min in PBS/5% normal goat serum and then incubated with the primary antibody in blocking buffer for 1 hour at room temperature. Immunofluorescence (IF) stainings were revealed by incubation with Alexa488 or Alexa568 labeled secondary antibodies (Molecular Probes) and nuclei were stained with DAPI (SIGMA). IHC stainings were evaluated on an AxioVert microscope (Zeiss, Germany) and IF stainings on a Leica DMI 4000 microscope (Leica Microsystems, Germany).

### Statistical Analysis

Statistical analysis and graphs were generated using the GraphPad Prism software (GraphPad Software Inc, San Diego, CA). All statistical analysis was performed by unpaired, two-sided t-test.

### Gene Expression Profiling Data

The raw data of gene expression profiling of Py2T cells in the absence and presence of TGFβ is publicly available at the ArrayExpress Database (Accession number E-MEXP-3731, available at http://www.ebi.ac.uk/arrayexpress/).

### Ethics Statement

Animal experiments were performed in strict accordance with the guidelines of the Swiss Federal Veterinary Office (SFVO) and the regulations of the Cantonal Veterinary Office of Basel-Stadt (license numbers 1878, 1907, and 1908). During the whole course of animal experiments, all efforts were made to minimize suffering.

## Supporting Information

Figure S1 **Canonical TGFβ signaling in untreated versus TGFβ-treated Py2T cells. (A)** Immunofluorescence staining for total Smad2/3 *(red)* and phosphorylated (activated) pSmad3 *(green).* Nuclei are visualized by DAPI staining. Scale bar, 20 µm. **(B)** Transcriptional Smad activity was determined by a dual luciferase reporter assay. Cells were transfected with a Smad4 luciferase reporter containing a Smad-binding element (SBE-luc) or a control plasmid lacking the SBE (luc), along with Renilla luciferase for normalization. Relative luminescence units (RLU) are expressed as mean +/− S.E.M from 2 independent experiments.
(TIF)

Figure S2 **Py2T tumors are characterized by a high immune cell infiltration.** Immunofluorescence staining of a Py2T tumor for the leukocyte marker CD45 and the macrophage marker F4/80. Images show a central region of a tumor grown in nude mice as described in Figure 6. Scale bar, 50 µm.
(TIF)

Figure S3 **Expression of a dominant-negative TGFβ receptor prevents EMT** ***in vitro*** **and does not significantly affect tumor growth. (A)** Py2T cells stably expressing a dominant-negative TGFβRII (Py2T TBRDN) or cells transduced with empty vector control were treated with TGFβ (2 ng/mL). To assess activation of canonical ΤGFβ signaling and nuclear accumulation of Smad proteins, cells were stained with an antibody against Smad2/3. To evaluate the breakdown of cell junctions downstream of TGFβ signaling, cells were stained with E-cadherin (adherens junctions) and ZO-1 (tight junctions). Scale bars, 50 µm. **(B)** Tumor growth of Py2T TBRDN and control cells (Experiment is described in Figure 6). n = 10 mice per group. Data is presented as mean ± S.E.M. Statistical values are calculated by using an unpaired, two-tailed t-test. A p-value >0.05 was considered not significant.
(TIF)

Figure S4 **Soft agar colony formation of epithelial and mesenchymal Py2T cells. (A)** Anchorage-independent growth of epithelial Py2T and mesenchymal Py2T LT cells. Cells were embedded in soft agar and supplemented with growth medium containing TGFβ (Py2T LT) or not (Py2T) and were allowed to grow for 10 days. **(B)** Quantification of formed colonies. Data is presented as mean ± S.E.M. Statistical values are calculated by using an unpaired, two-tailed t-test. ***p-value <0.001.
(TIF)

Movie S1 **Movie corresponding to** **Figure 4B:** **scratch wound closure of untreated Py2T cells for 13 days.**
(MOV)

Movie S2 **Movie corresponding to** **Figure 4B:** **scratch wound closure of Py2T cells treated with TGFβ for 13 days.**
(MOV)

Movie S3 **Movie corresponding to** **Figure 4C:** **Live imaging of Py2T cells grown on 2D tissue culture plastic.**
(MOV)

Movie S4 **Movie corresponding to** **Figure 4C:** **Live imaging of Py2T LT cells grown on 2D tissue culture plastic.**
(MOV)

Movie S5 **Movie corresponding to** **Figure 4D:** **Animation of Py2T cells grown in extracellular matrix and stained for either E-cadherin (** ***red*** **) and ZO-1 (** ***green*** **) or vimentin (** ***red*** **) and fibronectin (** ***green*** **), respectively.**
(MOV)

Movie S6 **Movie corresponding to** **Figure 4D:** **Animation of Py2T LT cells grown in extracellular matrix and stained for either E-cadherin (** ***red*** **) and ZO-1 (** ***green*** **) or vimentin (** ***red*** **) and fibronectin (** ***green*** **), respectively.**
(MOV)

Table S1 **List of genes that are significantly differentially expressed by at least 2 fold between Py2T cells (Py2T) and Py2T cells treated with TGFβ for 20 days (Py2T LT).** Data is derived from two independent experiments.
(XLS)

Table S2 **Sequences of RT-qPCR primers used.**
(DOC)
