# Supplementary figures and images for: Py2T Murine Breast Cancer Cells, a Versatile Model of TGFβ-Induced EMT In Vitro and In Vivo
Source: PLoS One. 2012 Nov 7;7(11):e48651. doi: 10.1371/journal.pone.0048651 (PMC3492491; doi:10.1371/journal.pone.0048651)

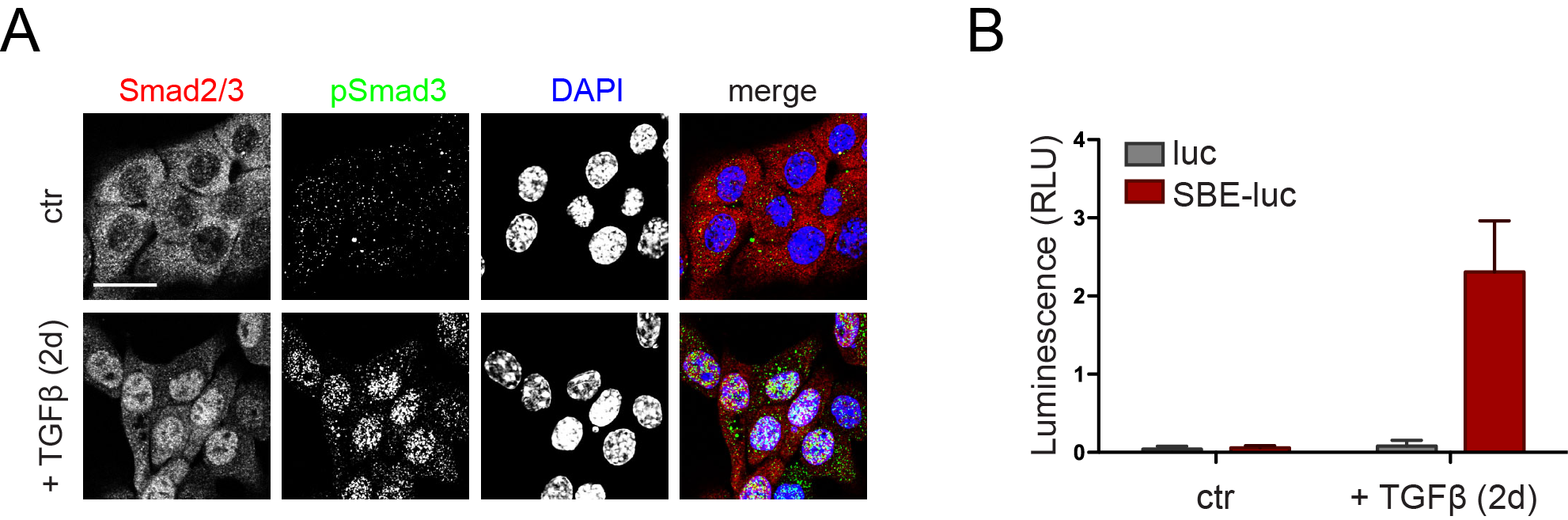

Supplement: Figure S1 — Canonical TGFβ signaling in untreated versus TGFβ-treated Py2T cells. (A) Immunofluorescence staining for total Smad2/3 (red) and phosphorylated (activated) pSmad3 (green). Nuclei are visualized by DAPI staining. Scale bar, 20 µm. (B) Transcriptional Smad activity was determined by a dual luciferase reporter assay. Cells were transfected with a Smad4 luciferase reporter containing a Smad-binding element (SBE-luc) or a control plasmid lacking the SBE (luc), along with Renilla luciferase for normalization. Relative luminescence units (RLU) are expressed as mean +/− S.E.M from 2 independent experiments. (TIF) [file pone.0048651.s001.tif]

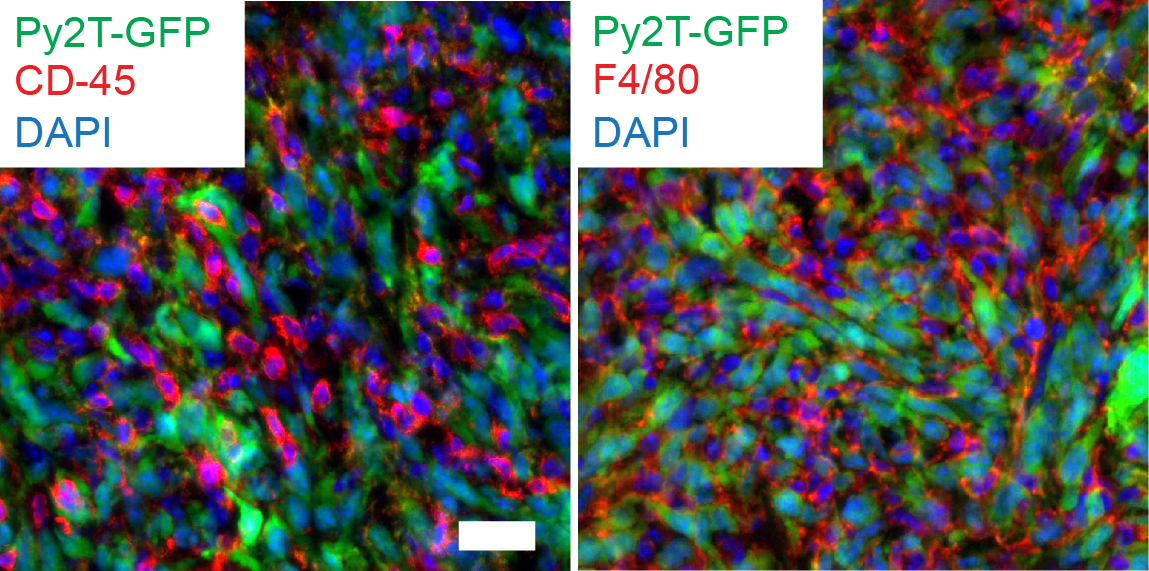

Supplement: Figure S2 — Py2T tumors are characterized by a high immune cell infiltration. Immunofluorescence staining of a Py2T tumor for the leukocyte marker CD45 and the macrophage marker F4/80. Images show a central region of a tumor grown in nude mice as described in Figure 6. Scale bar, 50 µm. (TIF) [file pone.0048651.s002.tif]

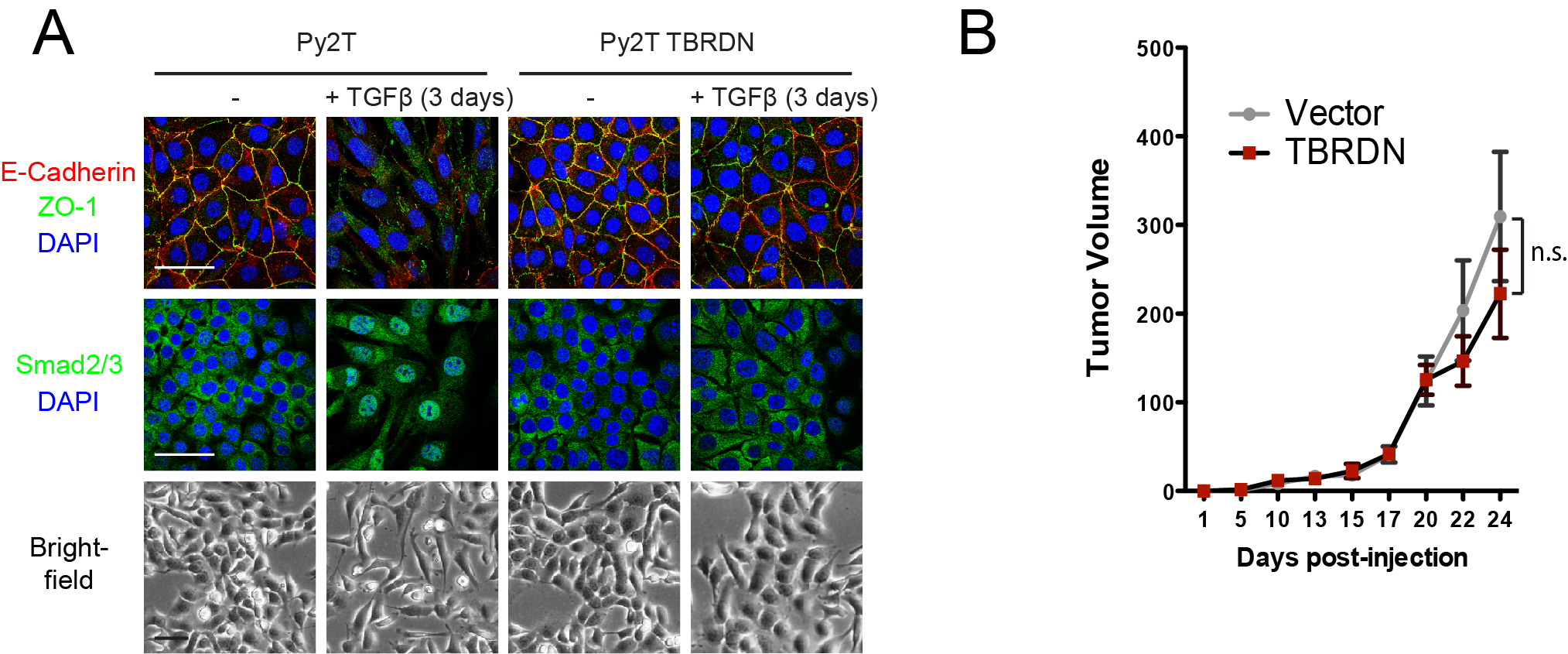

Supplement: Figure S3 — Expression of a dominant-negative TGFβ receptor prevents EMT in vitro and does not significantly affect tumor growth. (A) Py2T cells stably expressing a dominant-negative TGFβRII (Py2T TBRDN) or cells transduced with empty vector control were treated with TGFβ (2 ng/mL). To assess activation of canonical ΤGFβ signaling and nuclear accumulation of Smad proteins, cells were stained with an antibody against Smad2/3. To evaluate the breakdown of cell junctions downstream of TGFβ signaling, cells were stained with E-cadherin (adherens junctions) and ZO-1 (tight junctions). Scale bars, 50 µm. (B) Tumor growth of Py2T TBRDN and control cells (Experiment is described in Figure 6). n = 10 mice per group. Data is presented as mean ± S.E.M. Statistical values are calculated by using an unpaired, two-tailed t-test. A p-value >0.05 was considered not significant. (TIF) [file pone.0048651.s003.tif]

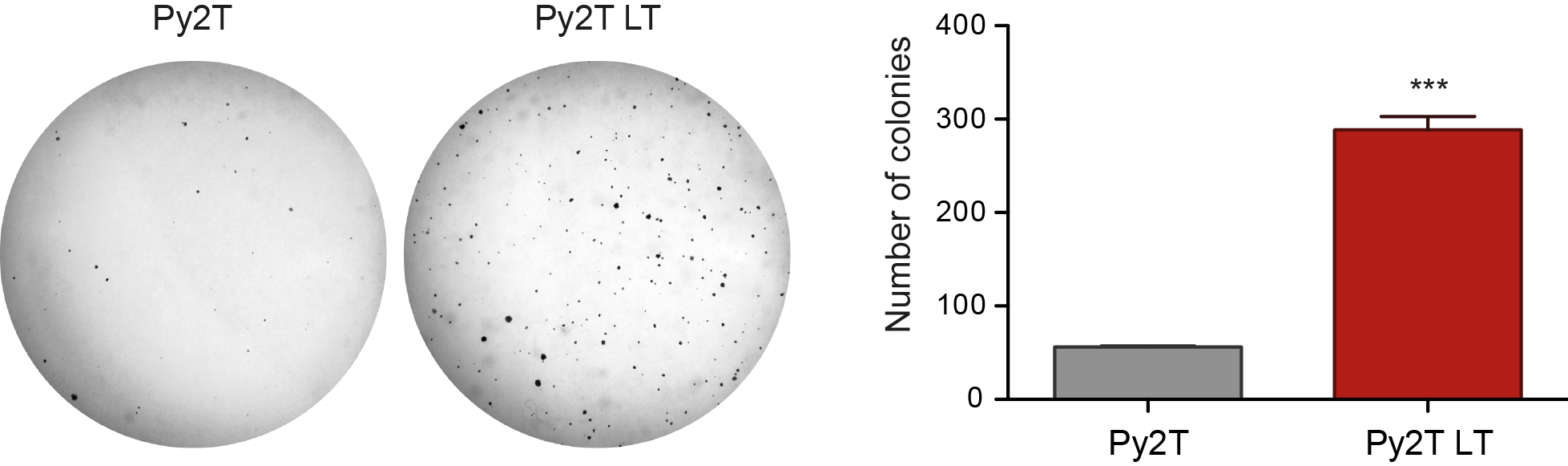

Supplement: Figure S4 — Soft agar colony formation of epithelial and mesenchymal Py2T cells. (A) Anchorage-independent growth of epithelial Py2T and mesenchymal Py2T LT cells. Cells were embedded in soft agar and supplemented with growth medium containing TGFβ (Py2T LT) or not (Py2T) and were allowed to grow for 10 days. (B) Quantification of formed colonies. Data is presented as mean ± S.E.M. Statistical values are calculated by using an unpaired, two-tailed t-test. ***p-value <0.001. (TIF) [file pone.0048651.s004.tif]
