# Supplementary material for: Py2T Murine Breast Cancer Cells, a Versatile Model of TGFβ-Induced EMT In Vitro and In Vivo
Source: PLoS One. 2012 Nov 7;7(11):e48651. doi: 10.1371/journal.pone.0048651 (PMC3492491; doi:10.1371/journal.pone.0048651)
Supplement: Table S2 — Sequences of RT-qPCR primers used. (DOC) [file pone.0048651.s012.doc]

**Supplemental Table II. Primers used for quantitative RT-PCR.**

| **mRNA** | **forward primer (5’-3’)** | **reverse primer (5’-3’)** |
| --- | --- | --- |
| Rpl19 | ctcgttgccggaaaaaca | tcatccaggtcaccttctca |
| E-cadherin | cgaccctgcctctgaatcc | tacacgctgggaaacatgagc |
| N-cadherin | ctgccatgactttctacggaga | caatgacgtccaccctgttct |
| Fibronectin | cccagacttatggtggcaatt | aatttccgcctcgagtctga |
| Vimentin | ccaaccttttcttccctgaa | ttgagtgggtgtcaaccaga |
|
| Zeb1 | gccagcagtcatgatgaaaa | tatcacaatacgggcaggtg |
| Zeb2 | ggaggaaaaacgtggtgaactat | gcaatgtgaagcttgtcctctt |
| Snail | ctctgaagatgcacatccgaa | ggcttctcaccagtgtgggt |
|
| Slug | tgtgtctgcaagatctgtggc | tccccagtgtgagttctaatgtg |
|
| E47 | ggacattaacgaggccttccg | tggggttcaggttgcgttct |
| Twist | gccggagacctagatgtcattg | cacgccctgattcttgtgaa |
|
